# Application of Copper–Sulfur Compound Electrode Materials in Supercapacitors

**DOI:** 10.3390/molecules29050977

**Published:** 2024-02-23

**Authors:** Junhua Lu, Hedong Jiang, Pingchun Guo, Jiake Li, Hua Zhu, Xueyun Fan, Liqun Huang, Jian Sun, Yanxiang Wang

**Affiliations:** School of Materials Science and Engineering, Jingdezhen Ceramic Institute, Jingdezhen 333403, China; 2120028018@stu.jcu.edu.cn (J.L.); hdjiang19@163.com (H.J.); guopingchun@jci.edu.cn (P.G.); lijiake@jcu.edu.cn (J.L.); zhuhua@jcu.edu.cn (H.Z.); hlq9832@126.com (L.H.); sunjian@jci.edu.cn (J.S.)

**Keywords:** supercapacitors, copper–sulfur compounds, electrochemical properties, specific capacitance, stability

## Abstract

Supercapacitors (SCs) are a novel type of energy storage device that exhibit features such as a short charging time, a long service life, excellent temperature characteristics, energy saving, and environmental protection. The capacitance of SCs depends on the electrode materials. Currently, carbon-based materials, transition metal oxides/hydroxides, and conductive polymers are widely used as electrode materials. However, the low specific capacitance of carbon-based materials, high cost of transition metal oxides/hydroxides, and poor cycling performance of conductive polymers as electrodes limit their applications. Copper–sulfur compounds used as electrode materials exhibit excellent electrical conductivity, a wide voltage range, high specific capacitance, diverse structures, and abundant copper reserves, and have been widely studied in catalysis, sensors, supercapacitors, solar cells, and other fields. This review summarizes the application of copper–sulfur compounds in SCs, details the research directions and development strategies of copper–sulfur compounds in SCs, and analyses and summarizes the research hotspots and outlook, so as to provide a reference and guidance for the use of copper–sulfur compounds.

## 1. Introduction

**Figure 1:**
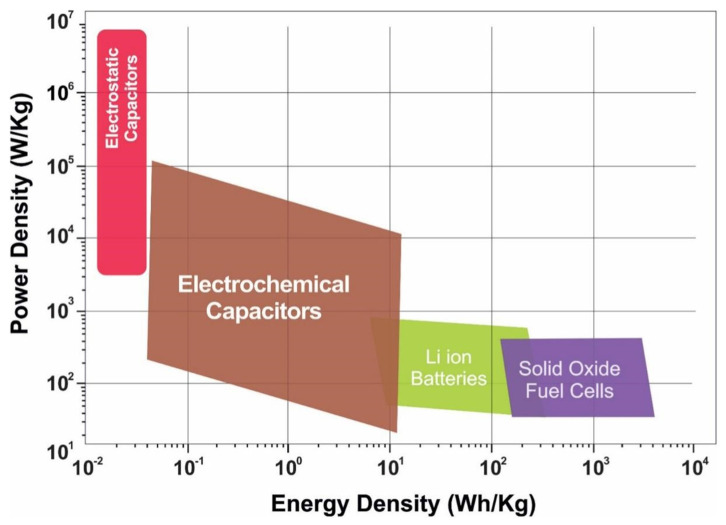
Ragone charts for various energy storage systems, including lithiumiom batteries, solid oxide fuel cells, electrostatic capacitors, and electrochemical capacitors [5].

To cope with the increasingly serious energy shortage, environmental pollution, and other related problems, researchers are vigorously developing green, efficient, and sustainable clean energy. With the rapid development of military equipment, aerospace, rail transit, new energy vehicles, power generation systems, and intelligent electronics, electrochemical energy storage devices have garnered considerable attention in recent years [1,2]. The current energy storage devices mainly include lithium-ion batteries, solid oxide fuel cells, electrostatic capacitors, and supercapacitors. Lithium-ion batteries have the advantages of having a high energy density, long life, low self-discharge rate, etc., and are currently the most common commercially used secondary batteries. However, lithium-ion batteries also have some disadvantages, such as their high cost, environmental sensitivity, and unnecessary heating due to the slow redox process, which can easily lead to thermal runaway and fire [3]. Solid oxide fuel cells offer benefits of being metal-free catalysts, having wide fuel sources, and cogeneration; however, the high reaction temperature of SOFCs leads to high maintenance costs and reduced battery durability over time. Each battery component is exposed to high temperatures, resulting in interface problems that degrade battery performance [4]. The capacitive behavior of electrostatic capacitors refers to the existence of capacitance between electrodes, which involves charging and discharging processes under the action of an electric field. The larger the capacitance, the lesser the power loss. This capacitive effect is generated by the accumulation of charge between the electrodes and does not involve the redox process of electrons and ions. In most batteries, redox reactions often occur, which involve the redox of electrons and ions to produce an electric current. These shortcomings have led researchers to search for electrochemical energy storage systems superior to existing batteries. An SC is an energy storage device based on high-speed electrostatic or Faraday electrochemical processes. Figure 1 presents the energy density and power density of various energy storage devices [5]. Compared with batteries, supercapacitors (SCs) exhibit a high theoretical energy efficiency of nearly 100%, which is conducive to the application of SC electrochemical devices in power grid load balancing [6]. In addition, SCs are new energy storage devices with a high power density, superior charging/discharging performance, low maintenance cost, safe operation, strong adaptability, good stability, and environmental friendliness, which can shorten the charging time from several hours to several minutes, improve the reliability of renewable power, and reduce waste [7,8,9].

**Figure 2:**
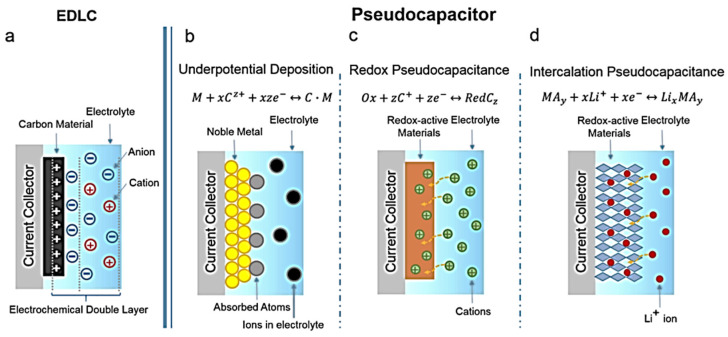
Schematics of charge storage mechanisms for (**a**) an EDLC and (**b**–**d**) different types of pseudocapacitive electrodes: (**b**) underpotential deposition, (**c**) redox pseudocapacitor, and (**d**) ion intercalation pseudocapacitor [11].

Electrode materials determine the efficiency of electrochemical energy storage systems, and depending on the energy storage mode, SCs can be divided into double-layer capacitors and pseudocapacitors. Electrode materials used for double-layer capacitors are mainly carbon-based materials (such as graphene) and some two-dimensional materials such as MoS_2_. These materials have a high power density; however, compared with pseudocapacitors, the energy density and specific capacitance of double-layer capacitors are low, and graphene sheets are prone to agglomeration, resulting in a decrease in the specific surface area, which eventually reduces the capacity. Graphene is often used as a skeleton material to compound with other materials. MoS_2_ has good specific capacitance; however, its electrochemical performance is limited by its inherent secondary agglomeration and low conductivity. In pseudocapacitors, energy is stored through the Faraday redox reaction. At the electrode and electrolyte interface, redox reactions result in higher specific capacitance, energy, and power density [10]. The electrochemical dynamics of pseudocapacitors are capacitive; however, charge storage is achieved through the charge transfer Faraday reaction across the double electric layer. The processes that derive from the Faraday process are fast and reversible surface redox thermodynamics, but capacitance is derived from the linear relationship between the degree of adsorbed charge and the change in potential. Charge storage in pseudocapacitors is generally divided into three types: underpotential deposition occurs at the two-dimensional metal and electrolyte interface, and ions are deposited at the metal interface when the potential is more positive than the corresponding reversible redox potential; redox pseudocapacitance occurs in the Faraday redox system; and in embedded/unembedded pseudocapacitors, ions are embedded in the redox active material but do not undergo crystalline phase transitions during the reaction, that is, their crystal structure does not change. Figure 2 [11] shows a schematic diagram of the charge storage mechanisms for double-layer capacitors and electrodes of different types of pseudocapacitors. Ther electrode materials used for pseudocapacitors are transition metal oxides, conductive polymers, and transition metal sulfides. Transition metal oxides (e.g., RuO_2_ and V_2_O_5_) have a high theoretical capacity, but poor conductivity leads to their low practical capacity; the voltage window is narrow and can only be applied in aqueous electrolytes [12,13]. The conductive polymer is accompanied by the doping/dedoping of ions during the energy storage process, leading to the repeated entry and exit of ions on the polymer chain, causing the fracture of the molecular chain as well as the generation of irreversible capacity, resulting in poor stability. However, transition metal sulfides have attracted the attention and interest of many researchers because of their low cost, better conductivity than oxides, high theoretical capacity, and especially, their high pseudocapacitance capacity. Currently, transition metal sulfides used in SCs mainly include Cu_x_S (x = 1–2), MoS_2_, Co_9_S_8_, NiS, Ni_3_S_2_, and WS_2_ [14]. In 2004, Stevic et al. [15] used copper–sulfur compounds as an electrode material for new SCs and achieved a capacitor capacity as high as 100 F cm^−2^. Copper–sulfur compounds exhibited a high electronic conductivity, large theoretical specific capacity, excellent redox reversibility, flat voltage plateau, excellent low temperature performance, tunable morphology and composition, rich copper reserves, low resistivity, and a lower electronegativity of sulfur than oxygen. Cu_x_S showed significant size-dependent electrochemical properties. Studies have shown that the change in morphology and the reduction in size affect the electrochemical characteristics of pseudocapacitors. Therefore, copper–sulfur compounds have great potential in SCs. This review highlights the current research status, directions, and development strategies of copper–sulfur compounds in SCs.

## 2. Crystal Structure and Properties of Copper–Sulfur Compounds

**Figure 3:**
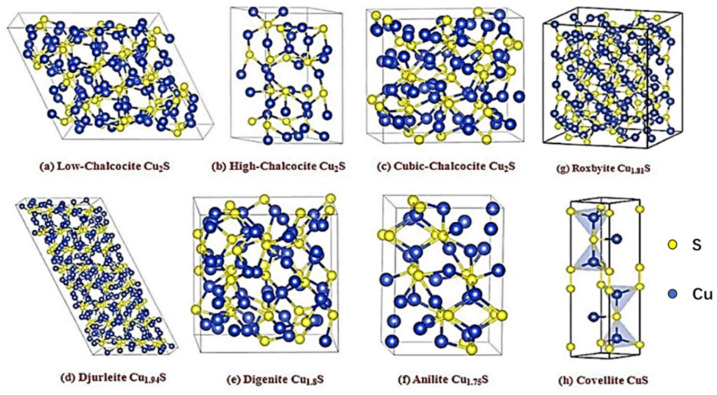
Crystal structure of Cu_x_S [16].

The crystal structure of materials is an important factor in designing energy storage materials. By adjusting the crystal structure and lattice defects, the conductivity and ion diffusion performance can be improved, so as to improve the charge and discharge efficiency of the energy storage material. In addition, the crystal structure can affect the stability and cycle life of the material, further improving the reliability of the energy storage material. Copper–sulfur compound Cu_x_S (1 ≤ x ≤ 2) with Cu vacancies is a p-type semiconductor with a forbidden bandwidth in the range 1.2–2.0 eV. Its controllable morphology, dimensions, crystalline phases, and compositions have facilitated its extensive application in photocatalysis, energy conversion, and biomedicine. In the Cu_x_S system, both stoichiometric solid solutions and a large number of nonstoichiometric phases exist. Even in the same crystalline phase, the morphology differs considerably. The crystal structure of the Cu_x_S system can be described as the stacking of S atoms in “hcp” or “fcc”, with the Cu atoms occupying distinct interstitial positions. In each crystal phase, all Cu atoms are not equivalent, and the interstitial spaces remain partially filled. Thus, numerous vacancy defects exist. Because of the small differences in atomic radii and electronegativity between Cu and S atoms, the s and p orbitals of the S atoms are coupled with the outer s and p orbitals of the Cu atoms, resulting in a pronounced covalency of Cu_x_S. Furthermore, S–S bonds form between S atoms and S atoms and Cu atoms and Cu atoms bond with each other inside Cu_x_S, which increase the complexity and diversity of the Cu_x_S structure. Cu_x_S has eight crystalline phases, namely low-chalcocite (monoclinic Cu_2.0_-_1.997_S), high-chalcocite (hexagonal Cu_2.0_-_1.94_S), cubic-chalcocite (Cu_2_S), djurleite (Cu_1.97_S-Cu_1.94_S), digenite (Cu_9_S_5_ or Cu_1.8_S), anilite (Cu_7_S_4_ or Cu_1.75_S), roxbyite (Cu_58_S_32_ and Cu_1.81_S), and covellite (CuS). Figure 3 displays the specific crystal structure of Cu_x_S [16]. Cu_2_S exhibits three phases, namely the α phase (>425 °C) shown in Figure 3c, the β phase (105–425 °C) shown in Figure 3b, and the γ phase (<105 °C) shown in Figure 3a [17]. Among these three phases, the two-dimensional *β*-Cu_2_S cell comprises S atoms that form a graphene-like hexagonal structure. Furthermore, γ-Cu_2_S is more stable than the other two phases and belongs to the monoclinic crystal system. The crystal structure of Cu_1.94_S (Figure 3d) is similar to that of γ-Cu_2_S, which also belongs to the monoclinic crystal system, and a single Cu_1.94_S cell can be written as Cu_62_S_32_, with 52 of the 62 Cu atoms located in triangular complexes formed by the S atoms, 9 on tetrahedra formed by the S atoms, and 1 in a linear complex. Cu_1.81_S is highly complex and belongs to the triclinic crystal system (Figure 3g), with 32 S atoms in the cell forming a densely arranged hexagonal structure, and 58 Cu atoms located in triangular complexes formed by S atoms at various interlayers. Cu_1.75_S is similar to *γ*-Cu_2_S, belonging to the monoclinic crystal system. Cu_1.75_S belongs to the orthorhombic crystal system (Figure 3f) where S atoms are nearly face-centered cubic, and Cu atoms are regularly arranged in the interstices of S atoms. A single crystal cell contains 28 Cu atoms, of which 20 are located in triangular fits and 8 in tetrahedra. CuS has a peculiar, layered structure. As displayed in Figure 3h, in each cell, four Cu atoms form a tetrahedral structure, two Cu atoms are located in the triangular coordination structure, two of the four S atoms form a disulfide structure, and the other two S atoms are individual S ions. Among these, CuS, Cu_1.75_S, Cu_1.8_S, and Cu_2_S are promising for energy storage applications because of their excellent crystal structure, electrical conductivity, high theoretical capacity (561, 305, 315, and 337 mAh g^−1^, respectively), and charging and discharging characteristics [18,19].

## 3. Copper–Sulfur Compounds with Different Stoichiometric Ratios in SC

**Figure 4:**
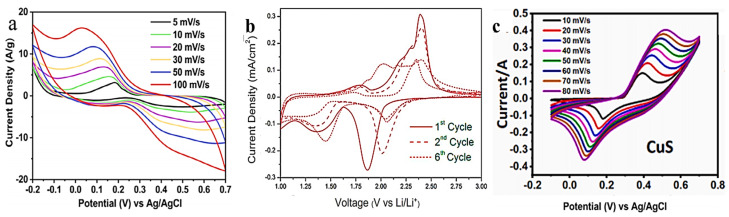
(**a**) CV curves of CuS in 1 M H_2_SO_4_ electrolyte at different scanning speeds; (**b**) CV curves of CuS in 1 M LiPF_6_ electrolyte at different scanning speeds; (**c**) CV curves of CuS in 3 M KOH electrolyte at different scanning speeds [23,24,29].

As shown in Figure 3h, Cu in CuS exists in two chemical environments, namely CuS_3_ with planar triangles and CuS_4_ with a tetrahedral structure. Therefore, the cell of CuS is connected in the form of a unit cell of CuS_4_–CuS_3_–CuS_4_, with each of the two-layer cells being interconnected by S–S bonds [20]. The unique layered structure of CuS can be used as an open channel to allow the embedding and de-embedding of ions through variations in S-S bond lengths and as an energy storage material. CuS exhibits a high electronic conductivity (10^−3^ S cm^−1^), high theoretical specific capacity (560 mAh g^−1^), an excellent crystal structure, and variable valence states, rendering CuS a potential contender for advanced energy storage applications [21]. The pseudocapacitance behavior of CuS electrodes is similar to that of capacitors because they contain a large number of charge/discharge holes and electron storage areas. With the increase in surface area, its size decreases, the diffusion path is shortened, the surface-based “ion storage site” is increased, and the pseudocapacitance behavior is improved. The performance of pseudocapacitance in a SC is affected by various factors such as the electrochemical window and electrolyte. The electrochemical window indicates the voltage span/range (V) over which the SC system (electrodes and electrolytes) is stable for chemical decomposition or irreversible conversion. It is an important parameter affecting the specific energy and specific power, which can be adjusted by appropriately selecting electrodes and electrolytes when designing SCs. In addition, the choice of electrolyte can affect the specific redox reaction in the pseudocapacitance of copper–sulfur compounds. Water-based electrolytes have gained popularity due to their easy availability, workability, and high ionic conductivity. They provide small protons with high ionic conductivity in an acidic aqueous electrolyte with better ionic diffusion. The redox peak of CuS in the CV curve of an acidic electrolyte is shown in Figure 4a. A pair of redox peaks of Cu^+^ and Cu^2+^ exist near 0.05 V and 0.4 V, corresponding to the mechanism of redox pseudocapacitance, as shown in Equation (1) [5,22]:

(1) $${Cu}^{+}=x{Cu}^{2+}+e^{-}$$

Although the commonly used acidic water electrolytes exhibit high ionic conductivity, a large number of charge and discharge cycles often lead to the dissolution of the electrode material. Foley et al. [23] studied the CV curves of CuS in the voltage range of 1.00–3.00 V at room temperature, as shown in Figure 4b. CuS/C was used as the electrode material to test its electrochemical performance in a 1 M LiPF_6_ electrolyte. Due to the insertion and extraction processes of Li^+^ ions, the peaks of reduction (1.85 and 1.35 V) and oxidation (1.8 and 2.4 V) reactions appeared in the first cycle of CuS. During the cycle of the two pairs of redox peaks in the CV curve, the redox reactions of CuS/CuSOH and Cu^2+^/Cu occur, expressed as Equations (2) and (3), respectively [23,24]:

(2) $$CuS+x{Li}^{+}+xe^{-}↔{Li}_{X}CuS$$

(3) $${Li}_{X}CuS+\left(2-x\right){Li}^{+}+\left(2-x\right)e^{-}↔{Li}_{2}S+Cu$$

In addition, alkaline electrolyte conditions are commonly used in copper–sulfur SC compounds. Shah et al. tested the CV curves of CuS at different scanning rates (10–80 mV s^−1^) under the potential window range of −0.1 to 0.7 V. Figure 4c shows a pair of redox peaks produced by Cu^2+^ and Cu^+^ with the potential of 0.5 V. As depicted, CuS has Faraday pseudocapacitance in a KOH electrolyte, and the corresponding electrode pseudocapacitance corresponds to Equations (4)–(7) [25,26,27,28,29,30,31]:

Reduction process:

(4) $$CuS+OH^{-}\to CuSOH+e^{-}$$

(5) $$CuSOH+OH^{-}\to CuSO+H_{2}O+e^{-}$$

Oxidation process:

(6) $$CuSO+H_{2}O+e^{-}\to CuSOH+OH^{-}$$

(7) $$CuSOH+e^{-}\to CuS+OH^{-}$$

**Figure 5:**
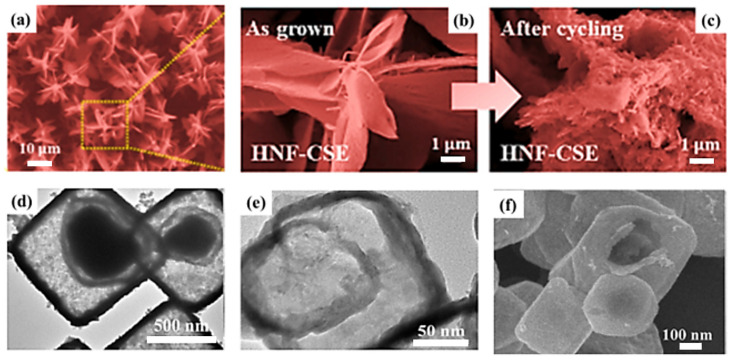
(**a**) Scanning electron microscopy (SEM) images of flower-like CuS; SEM images of CuS before (**b**) and after (**c**) the 20,000 charge/discharge cycle test; transmission electron microscopy (TEM) images (**d**,**e**) and SEM images (**f**) of CuS double-shell hollow nanocage structures [33,34].

Currently, various CuS morphologies such as nanoflowers, nanorods, nanosheets, and hollow cages for SC electrodes have been reported. Kim et al. [32] reported a hierarchical nanostructured flower-like CuS electrode consisting of a one-dimensional nanorod skeleton and two-dimensional CuS nanosheets. The material exhibits a high capacitance of 1.35 F cm^−2^ at a current density of 2 mA cm^−2^ and a capacity retention of 93.2% after 20,000 charge/discharge cycles. Figure 5 displays the scanning electron microscopy (SEM) images of the CuS electrode before and after cycling. No physical changes were observed even after 20,000 cycles, which reflects the high structural robustness and durability of the material, and the multistage structure indicated that the SC with the material as the electrode has excellent electrochemical properties. Heydari et al. [33] constructed CuS nanorods with a porous structure, with diameters of 90–110 nm and a specific surface area of 65 m^2^ g^−1^. The specific capacitance of the SC composed of these nanorods as electrodes was as high as 814 F g^−1^ at a current density of 1 A g^−1^. The capacitance retention rate of the electrode was as high as 95.9% after 5000 cycles. The remarkable electrochemical performance is attributed to its unique structural characteristics, which shorten the diffusion channels of ions and electrons, accelerate charge transport, and increase the density of the electroactive site during electron transfer. Guo J. et al. [34] prepared CuS with a hollow nanocage structure by using nanocubic Cu_2_O as a precursor through repeated vulcanization in the Na_2_S solution, followed by etching using hydrochloric acid. The SEM photos of CuS are shown in Figure 5d–f. The capacitor has a high specific capacitance of 843 F g^−1^ at a current density of 1 A g^−1^ and exhibits 89.2% capacitance retention after 4000 cycles of testing. The hollow nanocage structure provides a buffer space for the volume expansion caused by CuS during the charge and discharge processes, opening a channel for electrolyte penetration and storage, thus improving the cyclic stability of the capacitor. The characteristics of current supercapacitors with CuS as electrodes are listed in Table 1.

**Figure 6:**
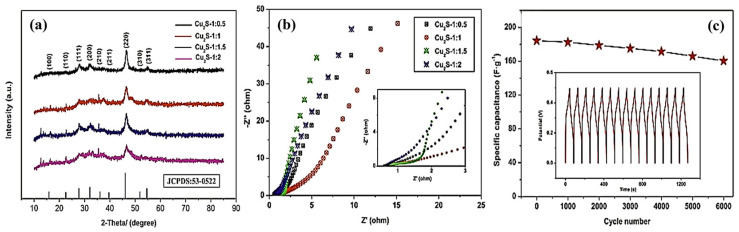
X−ray diffraction patterns (**a**) and Nyquist curves (**b**) at various ratios; (**c**) cycling performance of Cu_2_S formed at 1:1.5; the inset shows the charge/discharge curves [54].

**Figure 7:**
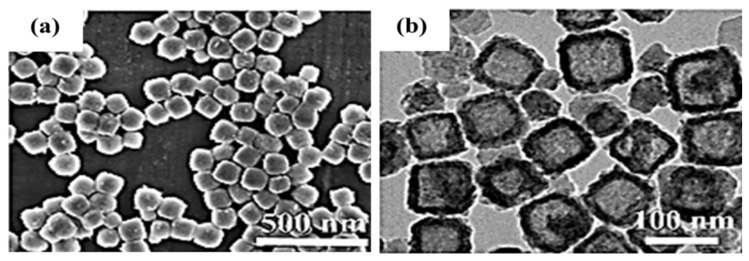
SEM (**a**) and TEM (**b**) images of Cu_2_S@NC [55].

In addition to CuS, Cu_2_S is widely used as a copper–sulfur compound. Cu_2_S has the highest copper content and can be used as an indirect bandgap semiconductor material and a direct bandgap semiconductor material with bandgaps of 1.2 and 1.8 eV, respectively. Its high theoretical specific capacity and high electrical conductivity render Cu_2_S a promising electrode material for lithium-ion batteries and a potential SC electrode material. Through solution combustion, Masoudpanah et al. [53] synthesized Cu_2_S powder, with Cu(NO_3_)_2_·3H_2_O as an oxidant and a mixture of thiourea and glycine as fuel. The powder consisted of nanosheets with a thickness of approximately 30 nm, which were randomly arranged in the form of flowers. The obtained Cu_2_S had a specific surface area of 18.24 m^2^ g^−1^, a predominantly mesoporous structure (8–10 nm), and a pore volume of 0.18 cm^3^ g^−1^, exhibiting a high specific capacitance of 677 F g^−1^ at a current density of 1 A g^−1^. The specific capacitance of 779 F g^−1^ was obtained at a scan rate of 10 mV s^−1^. The assembled asymmetric supercapacitor Cu_2_S/MnO_2_ exhibits a high energy density of 42.3 Wh kg^−1^ and a capacity retention rate of 86% after 6500 cycles at the current density of 3 A g^−1^. The high physicochemical stability and high reversibility of the redox reaction can be attributed to the following reasons: Cu_2_S sheets have a large chemically active site for redox reaction and charge storage, whereas thin and porous Cu_2_S nanosheets have a short charge transport distance, ensuring a fast charge/discharge process. Zhao et al. [54] prepared Cu_2_S microspheres in various ratios using CuSO_4_·5H_2_O and Na_2_S_2_O_3_·5H_2_O as copper and sulfur sources, respectively. When the copper–sulfur ratio is 1:1.5, the prepared Cu_2_S microspheres are uniformly distributed with a diameter range of 1.5–3 μm. The microspheres had some thin layers on the surface, which are conducive to the generation of more active sites, thereby improving capacitance performance. The performance is shown in Figure 6. The capacitance retention of Cu_2_S formed at 1:1.5 was 62.6% at a sweep rate in the range of 20–100 mV s^−1^, and the specific capacitance was 444.2 F g^−1^ at a current density of 1 A g^−1^. The capacity retention was 87% after 6000 cycles in a 6 M KOH electrolyte at 4 A g^−1^. Peng et al. [55] designed a uniform nitrogen-doped carbon-coated Cu_2_S hollow nanocube (Cu_2_S@NC) as an anode material (Figure 7.), which exhibited excellent cycling performance (317 mAh g^−1^ after 1200 cycles at 1 A g^−1^) and excellent multiplicative capacity (257 mAh g^−1^ at 6 A g^−1^) in a half cell. The carbon coating layer can prevent the aggregation of nano Cu_2_S sheets, the hollow nanostructure can shorten the ion/electron diffusion distance, and the inner cavity can buffer the volume expansion to facilitate adaptation to the volume change.

**Figure 8:**
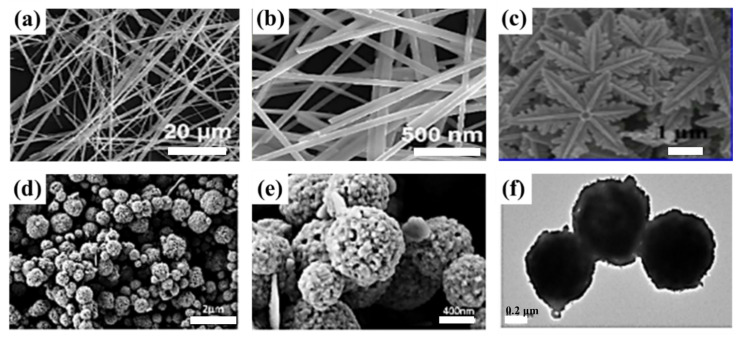
SEM images (**a**,**b**) of Cu_7_S_4_ nanowires; hexagonal snowflake-like Cu_7_S_4_ (**c**); SEM images (**d**,**e**) and TEM images (**f**) of porous submicrospheres Cu_7.2_S_4_ [56,57,58].

The theoretical specific capacitance of Cu_1.75_S is lower than those of CuS and Cu_2_S, and reports on the use of Cu_1.75_S as the SC electrode are scarce. According to the calculations, under Cu-rich conditions, Cu_1.75_S exhibits the lowest formation energy and is the most stable Cu_x_S. Moreover, the electrical conductivity of Cu_1.75_S is excellent due to the presence of Cu–Cu bonds in the lattice. Wang et al. [56] synthesized hexagonal snowflake-like nano-Cu_1.75_S with a diameter of 10 μm by using the hydrothermal method (Figure 8c), which exhibited a high specific capacitance of 1303.01 F g^−1^ at a current density of 5 A g^−1^. Javed et al. [57] synthesized highly conductive Cu_1.75_S nanowires by using a hydrothermal method at 200 °C and coated them on carbon fibers. The average diameter of Cu_1.75_S nanowires is approximately 100–150 nm (Figure 8a,b), and the specific surface area is approximately 34.23 m^2^ g^−1^, which is higher than those of CuS nanotubes (12 m^2^ g^−1^) and CuS nanoflowers (18.8 m^2^ g^−1^). The specific capacitance was 400 F g^−1^ at a scan rate of 10 mV s^−1^, the capacity retention was 95% after 5000 charge/discharge cycles at a constant current of 10 mA, and the charge transfer resistance increased by only 0.07 Ω before and after 5000 cycles. The maximum energy density reaches 35 wh kg^−1^ when the power density is 200 w kg^−1^. The high surface area of the nanowire structure increases the contact interface between the electrolyte and the active material, while it shortens the charge transfer path, which is conducive to charge transfer. The copper–sulfur compound is deposited directly on the carbon fiber without any lamination and binder, which reduces the resistance between the active substance and the electrolyte, thereby improving the performance of the SC.

Cu_1.8_S exhibits two crystalline phases, namely a low-temperature (<364 k) hexagonal phase and a high-temperature (>364 k) cubic phase. The cubic phase structure in Cu_1.8_S is a typical ionic crystal structure in which Cu and S atoms occupy canyon and vertex positions, respectively. Each Cu atom is surrounded by six S atoms in close proximity and each S atom is surrounded by three Cu atoms in close proximity, forming a stable crystal structure. The structure is highly ordered and closely packed. In addition, Cu ions form a face-centered cubic sublattice around the S ions, and as a superionic conductor, Cu ions have a mobility as high as in the molten state and in solution. The presence of Cu vacancies in Cu_1.8_S, with a bandgap of approximately 1.5 eV and abundant conductive holes in the energy bands, confers Cu_1.8_S with excellent electrical conductivity. Furthermore, Cu_1.8_S has a highly stable crystal structure. Because of the elemental abundance, low cost, and environmental friendliness of the compound, Cu_1.8_S exhibits considerable potential as a thermoelectric material with commercial applications. However, its theoretical specific capacitance is lower than those of CuS and Cu_2_S, which limits its applications in SCs. Zhou et al. [58] synthesized porous Cu_1.8_S with a submicroscopic spherical shape (Figure 8d–f), the specific surface area of 19.7 m^2^ g^−1^, and the electrode specific capacitance of 491.5 F g^−1^ (1 A g^−1^) by using the hydrothermal method with Cu_2_O as the precursor, and the specific capacitance retention rate was 491.5 F g^−1^ (1 A g^−1^) after 1000 cycles. Furthermore, after 1000 cycles, the specific capacitance retention rate was 82%, indicating that its energy storage performance and stability are excellent. The porous structure with a large specific surface area, three-dimensional multistage structure, and hollow nanostructure in different morphologies of copper–sulfur compounds are conducive to enlarging the chemical active site for charge storage in redox reactions, which shortens the diffusion distance of ions and electrons, enhances the charge transport efficiency, and expands the buffering volume, thus effectively improving the capacitance storage and cycle stability of copper–sulfur compound SCs.

## 4. Preparation of Copper–Sulfur Compounds for SC Applications

Copper–sulfur compounds are prepared using the hydrothermal, deposition, solvothermal, sacrificial template, continuous ionic layer adsorption, and sol–gel methods [17]. These methods are relatively simple in operation, low in cost, and high in productivity, and are often used to synthesize copper–sulfur compounds with different nanostructures. The morphology and structure of crystals determine their specific surface area and pore size distribution. These features are key factors affecting the number of active sites, electrolyte accessibility, transport/diffusion pathways, and kinetics of the electrochemical reactions at SC electrodes. Nanoporous structures with a high specific surface area and small pore size have attracted considerable research attention.

In the hydrothermal method, copper salts and vulcanizing agents are allowed to react in the aqueous phase at high temperatures and pressure to produce copper–sulfur compounds [59]. In the hydrothermal preparation of CuS, CuS nanoparticles are uniformly distributed and the morphology is regular, which is conducive to the electrochemical performance of the capacitor. Zhai et al. [60] used the hydrothermal method with CuSO_4_·5H_2_O and thiourea as raw materials to synthesize CuS with a 3D flower-like microsphere structure. Because of the high surface energy, nanosheets with thicknesses ranging from 20 to 40 nm aggregated to form stable flower-like microspheres with diameters of 4–6 μm, reducing the free energy. The microspheres exhibited a capacitance of 4176 mF cm^−2^ at a current density of 2 mA cm^−2^ with a capacitance retention of 84.9% after 5000 cycles. Ding et al. [61] prepared Cu_2_S with aggregate, microporous, and nanoparticle morphology in different reaction solvents by using the hydrothermal method. After 3000 constant current charge and discharge cycles, the capacitance retention rates were 93.7%, 94.1%, and 97%, respectively. Cu_2_S nanoparticles exhibit high structural stability under a high current, and through continuous reversible reaction, the active substance on the surface is continuously separated, thus increasing the specific surface area and the specific capacity of the material, eventually improving the retention rate significantly. The solvothermal method was developed based on the hydrothermal method, in which the original mixture is allowed to react in a closed system such as an autoclave with organic or nonaqueous solvents at a certain temperature and self-generated pressure of the solution. Zhou et al. [62] prepared hexagonal CuS nanoparticles by using the solvothermal method by combining CuCl_2_ and Na_2_S at 140 °C. The grain size of the nanoparticles was 77.6 nm, with specific surface area of 39.2 m^2^ g^−1^ and the pore size distribution of 9.58 nm. Their specific capacitance was 656.8 F g^−1^ at 1 A g^−1^, and the capacitance was maintained at 92.8% after 5000 constant current charge/discharge cycles. Kumar et al. [63] synthesized three-dimensional Cu_1.8_S with a nanoflower-like structure by the solvothermal method and showed the highest specific capacitance of 1050.0 F g^−1^ at a current density of 1 A g^−1^. Liu Y. [64] used the solvothermal method and adjusted solvents to different CuS hierarchies, including tubular, microsphere, and hollow flower CuS. Among these forms, the hollow flower CuS consists of nanosheets with an average diameter of approximately 150 nm and a large number of voids and exhibits the best electrochemical performance with a capacitance of 717.4 F g^−1^ at a current density of 1 A g^−1^, which is higher than those of the tubular CuS (597.0 F g^−1^) and microsphere CuS (451.6 F g^−1^). The capacitance was maintained at 83.6% for 20,000 consecutive cycles at a current density of 5 A g^−1^. The hollow flower-like structure allows ions and electrons to diffuse more easily, which is conducive to the electrode/electrolyte contact, shortening the transfer pathway of ions and electrons, and providing more active sites for maximum utilization. Hollow structures have attracted considerable attention due to their low density, clear walls, other structure-related characteristics, and application prospects in various fields [65].

**Figure 9:**
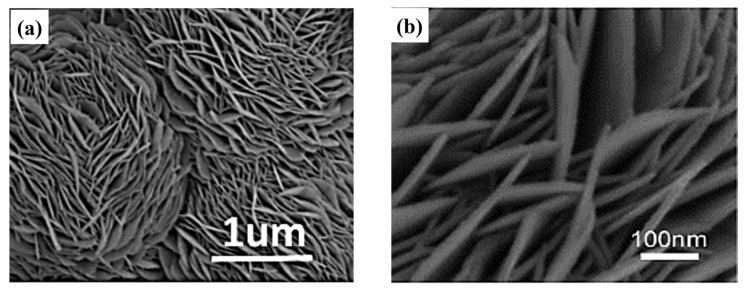
(**a**,**b**) SEM images of CuS at low and high magnification [67].

The chemical bath deposition method is used to deposit metals or other compounds on the surface of an object. Lokhande et al. [66] used this method to synthesize CuS with the nanoflower structure. The nanoflower-like morphology of the CuS electrode exhibited a specific capacitance of 1818.2 F g^−1^ at a scan rate of 5 mV s^−1^ and a capacity retention of 92% after 2000 cycles. CuS with a nanoflower structure has a large surface area (150.6 m^2^ g^−1^) and a small pore size (2.15 nm) distribution, providing more active sites and thus excellent electrochemical properties. Co-precipitation is a critical method for preparing ultrafine powders of composite oxides containing two or more metal elements, which have two or more cations that are present in the solution as homogeneous phases. A homogeneous precipitate of various compositions can be obtained by adding a precipitating agent in a precipitation reaction. Kajal et al. [67] used the co-precipitation method to prepare nanostructured CuS by varying the amount of the copper precursor. Using CH_4_N_2_S as the sulfur source, they synthesized two morphologies of CuS. The first type comprised microspheres aggregated from nanoparticles (grain size, 8.26 nm) using Cu(NO_3_)_2_·3H_2_O as the copper precursor, whereas irregular CuS nanosheets were obtained using CuCl_2_·2H_2_O as the copper precursor (Figure 9). The specific capacitance value was 346.6 F g^−1^ at a current density of 2 A g^−1^, and the constant capacitance retention was approximately 81.6% after 500 cycles at a current density of 10 A g^−1^.

## 5. Different Conductive Substrates for the Application of Copper–Sulfur Compounds in SC

Conductive substrates play crucial roles in the growth process of copper–sulfur compounds. First, they provide an excellent physical platform for the CuS compounds to achieve superior adhesion on the substrate and improve the overall electrical conductivity. This phenomenon is conducive to the growth of crystals. Second, the lattice structure, surface energy, morphology, and other parameters on the surface of the conductive substrates affect the growth rate, orientation, lattice parameters, and other aspects of the copper–sulfur compounds. Therefore, by choosing a suitable conductive substrate and controlling the growth conditions, the characteristics of copper–sulfur compounds such as morphology, structure, and crystal quality can be regulated. Patil et al. [68] precipitated CuS nanoparticles directly on a flexible stainless steel plate and obtained spherical and elliptical nanoparticles uniformly distributed on the surface of the film with low resistive values (Rs = 0.9 Ω, Rct = 3.96 Ω). The specific capacitance was 885 F g^−1^ at a sweep rate of 5 mV s^−1^ and 812 F g^−1^ after 2000 cycles, with a capacity retention of 92% (only 8% capacitance loss). Ying et al. [69] used ultrasound-assisted filling and chemical bath surface vulcanization on 3D hierarchically graded Cu-powder-filled nickel foams constructed with CuS nanoflowers. These composites had an ultra-high area capacitance of 11.4 F cm^−2^ at a high current density of 90 mA cm^−2^, and the capacitance retention was 85% after 3000 cycles at 180 mA cm^−2^.

To obtain high-quality electrodes, the bonding between the active material and the collector was increased without an additional filler/conducting agent. The performance of the SC was improved using copper as a conductive substrate. He et al. [70] grew 3D hollow tubular Cu_2_S nanorods (Cu_2_S@Cu) in situ on a copper foam by using sulfide. The copper substrate functioned as both a copper source and a collector, reducing the contact resistance (0.45 Ω). In addition, a high surface capacitance of 1000 mF cm^−2^ was obtained at a current density of 2 mA cm^−2^, and the capacitance retention reached 96.9% after 10,000 cycles. The SC exhibited excellent flexibility with a capacity retention of 99.8% and 86.1% under twisted and bent conditions, respectively. Lee et al. [71] grew CuS–NWs directly on copper wires by using liquid–solid chemical oxidation and anion exchange methods. The CuS–NWs consisted of grains of approximately 5–6 nm in size, with a facet spacing of 0.28 nm, a large pore volume of 0.2879 cm^3^ g^−1^, and a specific surface area of 102.8 m^2^ g^−1^. The CV confined area of CuS–NWs was approximately 2.3 times that of Cu(OH)_2_–NWs, and the face capacitance of CuS–NWs (378.0 mF cm^−2^) was 2.2 times that of Cu(OH)_2_–NWs (172.4 mF cm^−2^) at a current density of 2 mA cm^−2^. The capacitance retention of the CuS–NWs base electrode was 90.2% after 2000 charge/discharge cycles at a current density of 10 mA cm^−2^. Copper used as a conductive substrate serves as both a copper source and a collector, eliminating the need for additional adhesives and reducing the contact resistance, which results in the formation of a highly ordered three-dimensional structure, enlarged redox-reaction active sites, increased ion transport, and enhanced electrochemical performance.

## 6. Copper–Sulfur Composite with Other Materials in SC

The capacitance value of a copper–sulfur compound electrode material varies greatly with changes in the composition, shape, and structure. High agglomeration of copper–sulfur compound nanomaterials tends to reduce the effective surface area and thus the capacitance. The regulation of the morphology, porosity, and other properties of copper–sulfur compounds is random and cannot be accurately controlled as per the need, and inseparable mixed phases are easily generated [72]. In longer and faster charge and discharge cycles, the electrochemical stability of copper–sulfur compounds is low, and the volume change is irreversible, resulting in poor cycle efficiency [73,74]. Therefore, to effectively circumvent these challenges and achieve better electrochemical performance of copper–sulfur compound SCs, research on copper–sulfur compound composites with other materials is needed. The combination of copper–sulfur compounds with other materials can achieve synergies to further improve energy storage performance. The following are some possible synergies: (1) Conductivity enhancement: a composite with conductive materials (such as carbon materials, metal materials, or conductive polymers) can provide more electronic channels, enhance the conductive properties of the electrode material, and thus improve the charge transfer rate and electrode reaction speed. (2) Cycle stability enhancement: carbon materials have good cycle stability and structural stability, and the combination of copper–sulfur compounds can provide high cycle stability and a long service life, reducing material shedding and electrolyte impregnation problems. (3) Improved structural stability: through the composite with structural support materials (such as carbon nanotubes, graphene, etc.), the sulfide nanoparticles can be effectively fixed, reducing the problems caused by volume expansion, and providing a better charge transport path. (4) Specific capacity increase: by combining with high specific capacity materials (such as conductive polymers), the copper–sulfur compound/high specific capacity material composite electrode can achieve an increase in specific capacity and additional energy storage.

### 6.1. Forming Polymetallic Sulfides with Other Metals

Compared with mono-metallic sulfides, polymetallic sulfides increase the specific surface area, conductivity, and porosity, which result in abundant oxygen reduction reaction sites. Furthermore, combining various metal ions facilitates the synergistic effects of various metals, enabling multiple transition metal ions to participate in the redox reaction, which improves the specific capacitance and electrical conductivity of the metal sulfides [75,76]. The addition of other metals to copper sulfide compounds can overcome the disadvantages of single-phase materials such as insufficient redox activity, poor electrochemical behavior, low conductivity, and short cycle life, and improve material properties. Metals generally compounded with copper–sulfur compounds include cobalt, nickel, molybdenum, tin, and zinc.

Copper cobalt polysulfides exhibit high charge carrier mobility and compositionally and morphologically tunable stoichiometric tailoring capabilities [51,77]. Cobalt ions can provide abundant oxidized reduced valence states and improve electrochemical performance compared with other compound semiconductors [78,79]. Chavan et al. [80] prepared high-performance CoCuS thin films through chemical deposition. The surface of the films exhibited a flower-like wrinkled structure, with Co, Cu, and S uniformly dispersed on the film surface. The specific capacitance was 928 F g^−1^ at 5 mV s^−1^ with 88.82% Coulombic efficiency and 90% capacity retention after 5000 cycles in a 1 M KOH electrolyte. Furthermore, 10% capacitance loss was attributed to the dissolution or detachment of the active material. Wang et al. [81] synthesized hollow rhombic cage dodecahedral CuCo_2_S_4_ composites by using the solvothermal method. The cage has a high specific capacitance of 1096.27 F g^−1^ at 0.5 A g^−1^. The asymmetric supercapacitor, ASC, assembled with CuCo_2_S_4_ and AC provided an energy density of 87.59 Wh kg^−1^ at a power density of 399.65 W kg^−1^ and exhibited 95.83% capacity retention after 5000 cycles. The hollow cage alleviates the structural damage caused by the volume expansion during the Faraday redox reaction and improves the electrochemical performance of SCs. Zhao Y. et al. [82] proposed a two-step synthesis method involving refluxing and solvothermal methods for the template-derived mesoporous CuCo_2_S_4_ nanoflower by ZIF-67 composites. At a current density of 1 A g^−1^, a specific capacity of up to 1344 F g^−1^ is shown. Molybdenum exhibits high electrical conductivity, which can improve the electrical conductivity of electrode materials, promote rapid electron transfer and charge storage, increase the effective surface area of electrode materials, increase the capacitance of capacitors, improve the structural and chemical stability of electrode materials, and reduce the penetration of the electrolyte and side reactions with electrodes. Choi et al. [83] used a hydrothermal method to prepare CuMoS composites, consisting of nanospheres interconnected with each other to form a porous structure to promote charge transfer. The synthesized composites exhibited the specific capacitance of 3.5 F cm^−2^ at a current density of 3 mA cm^−2^, and its capacitance retention after 4000 cycles was 86.9%. Tin exhibits a low melting point as well as excellent plasticity and conductivity. The introduction of an appropriate amount of tin into copper sulfide electrode materials enhances the conductivity, improves the cycling stability, and increases the specific capacity. Yu et al. [84] synthesized floral Cu_5_Sn_2_S_7_ nanomaterials by using a one-step hydrothermal method. Its specific capacitance increased from 111 to 200 F g^−1^ at a current density of 1 A g^−1^, and it was maintained at 170 F g^−1^ even when the current density was increased to 10 A g^−1^, which provided excellent multiplicative performance. The capacity of 70.6% was observed after 3000 cycles of charging and discharging. The bimetal-S bond provides abundant redox active sites and high electrical conductivity, thereby improving the electrochemical performance of SCs. Sc-related data for polymetallic sulfide complex sites formed by copper-sulfur compounds with other metals are listed in Table 2.

### 6.2. Complexes with Other Metal Compounds

**Figure 10:**
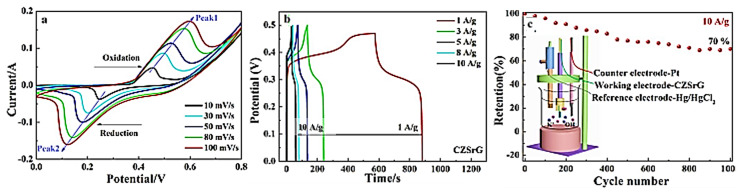
(**a**) Cyclic voltammetry (CV) curves of CZSrG at various scan rates; (**b**) galvanostatic charge/discharge (GCD) curves of CZSrG at various current densities; (**c**) cycling stability of CZSrG-3 at a current density of 10 A g^−1^ [98].

In electrodes composed of mixed metal sulfides, different microstructures can be obtained to provide rich redox reactions and synergistic effects on the SC. Nickel sulfide Ni_3_S_4_, as an electrode material, exhibits high specific capacity and excellent cycling stability, and the introduction of nickel sulfide into copper sulfide compound electrode materials can improve the electrochemical performance of the SC. Yue X. et al. [96] grew flower-like CuS microspheres on nanoporous copper and prepared three-dimensional spherical CuS/NiS by using the hydrothermal method. Each flower-like microsphere was formed by stacking many nanosheets ranging in thickness from 17 to 52 nm, and at the current density of 1 A g^−1^, the specific capacitance of 122 F g^−1^ could be achieved, whereas a capacitance retention of 95% after 5000 cycles was achieved at 10 A g^−1^. MoS_2_ nanosheets were used as a conductive package to protect the internal active material from damage during charging/discharging and improve the structural integrity and stability of the material through three-dimensional conductive cladding. Dai et al. [97] used hollow CuS nanocages on polyaniline (PANI) nanolayers coated on the surface. Subsequently, they prepared a MoS_2_ conductive cladding layer by using a hydrothermal method, and the CuS/PANI@MoS_2_ composites were developed. PANI wrapped around the framework of the CuS nanocages improved the stability of the composites. The nanosheets in the CuS/PANI@MoS_2_ composites exhibited pleats and defects, which not only increased the electrode material electrolyte contact area but also enhanced ion transport and improved electrolyte ion migration and electron transfer. The specific capacitance was 759.2 F g^−1^ at 1 A g^−1^ and the capacitance retention was 92.1% after 6000 cycles. ZnS as a p-type semiconductor is highly conductive and electrochemically active because of its small bandgap. Combining CuS/ZnS with conductive carbon materials to form composites can effectively solve the problems of low conductivity and aggregation. Furthermore, hydrogel electrodes exhibit the most beneficial effects by preventing considerable volume changes and increasing cycling stability. You et al. [98] prepared CuS/ZnS/sodium alginate/reduced graphene oxide hydrogel (CZSrG) hybrid electrode materials through physical crosslinking and by using the one-step reduction method. The three-dimensional mesh structure of the hydrogel-wrapped CuS/ZnS accommodated the volume change of CuS/ZnS and facilitated OH- and electron transport. An excellent specific capacitance of 992 F g^−1^ was achieved at a scan rate of 10 mV s^−1^. In this case, 70% of the initial specific capacitance was maintained even after 1000 charge/discharge cycles, as displayed in Figure 10c. The supercapacitor prepared from this composite had a specific capacitance of 252.1 F g^−1^ (5 mV s^−1^) and a power density of 1800 Wh kg^−1^ at an energy density of 2.05 Wh kg^−1^.

In addition to metal sulfides, transition metal hydroxides can be added to Cu_x_S SCs to enhance their performance. Transition metal hydroxides have high theoretical specific capacitance; for example, nickel hydroxide has a theoretical specific capacitance of 2382 F g^−1^. Transition hydroxides have an interlayer structure such as graphite, which enables their embedding in the electrolyte ions, thus allowing more ions to participate in the pseudocapacitive reaction and ensuring excellent electrochemistry. Zhou et al. [99] prepared core–shell-structured Cu_7_S_4_/Ni(OH)_2_ composites and ultrathin Ni(OH)_2_ composites. Ni(OH)_2_ composites, with ultrathin Ni(OH)_2_ nanosheets as “shells” anchored on Cu_7_S_4_ nanorods as “cores”, and binder-free core–shell hybrid nano-arrays on copper foam exhibited the specific capacitance of 1072.5 F g^−1^ at a current density of 1 A g^−1^. At a current density of 1 A g^−1^, the composites displayed a high specific capacitance of 1072.5 F g^−1^ with 94.5% capacitance retention after 10,000 cycles. The addition of copper hydroxide can improve the capacity and stability of the capacitor. Copper hydroxides exhibit an excellent ability to adsorb ions in the electrolyte, increasing the electrode surface area and improving the capacity of the capacitor; they also exhibit high chemical stability and corrosion resistance, which confer them long-term stability in complex environments. Sun M. et al. [100] synthesized porous Cu(OH)_2_/Cu_7_S_4_ hetero nanowires using the sacrificial template method at room temperature. Accelerating the transport of electrons and ions along their longitudinal direction reduces the electrical resistance (internal resistance Rs = 0.86 Ω and charge transfer resistance Rct = 1.99 Ω). The porous nanostructures not only exhibit a large surface area (102.8 m^2^ g^−1^) and pore volume (0.2879 cm^3^ g^−1^), short ion transport distances, and many channels for rapid electrolyte ion transport, but can also withstand large volume changes during charging and discharging, which is favorable to the cycling stability of SCs. Cu(OH)_2_/Cu_7_S_4_ nanowires coated on nickel foam were used as active electrode materials, which provided a high specific capacity of 1610.8 C g^−1^ at a current density of 4 A g^−1^ and that of 765.0 C g^−1^ at a higher current density of 30 A g^−1^.

Transition metal oxides can cause the ions in the electrolyte to undergo rapid redox reactions during pseudocapacitive reactions. Furthermore, they have a high theoretical specific capacity, superior to that of carbon-based materials, which is in the range 200–400 F g^−1^. Copper oxides exhibit high chemical stability and corrosion resistance, which are conducive to the capacity and stability of the capacitor. Wang et al. [101] prepared CuO/Cu_x_S_y_ octahedral composites with a double-shell layer hollow structure at room temperature by using the ion exchange method. Their double-shell layer consists of intersecting Cu_x_S_y_ nanosheets, with the inner core layer consisting of CuO. The unique structure and the synergistic effect of the two contribute to improving the electrochemical properties. The morphology and composition of the composites were influenced by the vulcanization time. The specific capacitances at 1 A g^−1^ current density were 132.6, 236, 413.6, 330.9, and 263.3 F g^−1^ at vulcanization times of 2, 4, 6, 8, and 12 h. With the increase in the reaction time, the specific capacitance first gradually increased and electrochemical performance improved because of more electrochemically active sites of sulfide compared with oxide, subsequently reaching the optimum level. When the vulcanization time was further increased, the thickness increased because of the increase in the CuO content, which led to the collapse of the hollow octahedral structure of the double-shell layer, resulting in the decrease in both the specific surface area and number of active sites, and deteriorated the electrochemical performance of the material. Tian et al. [102] prepared spherical NiCo_2_O_4_/CuS composites by using the hydrothermal method. The specific surface area of the composites was 106.2 m^2^ g^−1^ and their pore volume was 1.92 cm^3^ g^−1^, with a high specific capacitance of 2169 F g^−1^ at 0.5 A g^−1^. The asymmetric supercapacitor NiCo_2_O_4_/CuS/GO exhibited a high energy density of 173.5 Wh kg^−1^ at a power density of 360 W kg^−1^, which could be attributed to the synergistic effect of CuS and NiCo_2_O_4_, resulting in increased electron transport and electrolyte ion diffusion. Sc-related data on the complexation of copper-sulfur compounds with other metal compounds are presented in Table 3.

### 6.3. Copper–Sulfur Composite with Carbon-Based Materials for SC Applications

Carbon-based materials and their composites, such as graphene, carbon nanotubes, activated carbon, and acetylene black, have attracted considerable research attention in the energy field. These materials exhibit excellent electrochemical performance through the charge storage property of the bilayer behavior and are excellent SC-active electrode materials. Pure copper–sulfur compounds are semiconductors, and their conductivity is lower than those carbon nanomaterials, and compounding copper–sulfur compounds with carbon-based materials can produce more surface active sites to enhance redox reaction efficiency and pseudocapacitance and increase the cycling stability of the capacitor to enhance battery performance [5]. Moreover, the combination of copper–sulfur compounds with carbon-based materials including carbon coating as well as carbon nanotube encapsulation, graphene encapsulation, and core–shell structure formation reduce the agglomeration and cycle life of SCs due to the volume change of copper–sulfur compounds in the constant current charge/discharge process; however, it improves the electrochemical performance of SCs.

#### 6.3.1. Copper–Sulfur Composite with Graphene for SC Applications

Graphene exhibits excellent electrical conductivity as well as mechanical properties because of its unique honeycomb structure and large specific surface area (~2630 m^2^ g^−1^) with excellent ion diffusion paths and reduced diffusion resistance. Theoretically, the specific gravity capacitance of single-layer graphene is close to 500 F g^−1^, and the surface capacitance of its total surface area is 21 µF cm^−2^. Graphene is oxidized to hydrophilic GO, and the graphite layer spacing is increased from 3.35 Å prior to the oxidation to 7–10 Å after oxidation. The introduction of oxygen atoms in the oxidation process resulted in the formation of a large number of oxygen functional groups, which in turn resulted in a high surface area and many pores; however, the electrical conductivity decreased [109]. By contrast, reduced graphene (rGO) removes the oxygen functional groups and restores the honeycomb two-dimensional structure and high electrical conductivity of graphene. Graphene is typically combined with other materials by using two composite methods, namely surface growth and cladding [110]. Direct growth of a material on the surface of graphene can maintain its high conductivity and two-dimensional properties, which are favorable for electron transport. The preparation process of cladding is simple and can help in achieving a large area coverage, which is suitable for mass production. However, this method affects the two-dimensional properties of graphene and thus deteriorates the electron transport performance.

Currently, CuS is prepared using the hydrothermal method to form copper sulfides on the graphene surface, which controls the specific surface area, copper sulfide morphology (mainly nanosheets, nanorods, quantum dots, hexagonal grains, and nanoparticles), and microstructure of the electrodes to enhance the electrochemical performance of composites. Balu et al. [111] used a hydrothermal method to prepare CuS/GO. Woolly spherical CuS consisting of ultrathin CuS nanosheets uniformly modified on the graphene surface had a specific surface area of 40.3 m^2^ g^−1^, which is nearly twice compared with that of CuS nanospheres (20.8 m^2^ g^−1^). The average pore size of CuS/GO increased from 2.8 nm in the case of CuS nanospheres to 5.1 nm. At a sweep rate of 5 mV s^−1^, CuS/GO exhibited a specific capacitance of 197.45 F g^−1^ and a capacity retention of 90.35% after 1000 cycles at a current density of 5 A g^−1^. The unique nanorod structure embedded in the graphene network provides CuS/GO with a mesoporous structure, high surface area, and high electrical conductivity, which enlarges the interfacial area of the nanocomposites, facilitates electron transfer and electrolyte diffusion, and promotes the generation of more active sites in redox reactions to improve the electrochemical performance of SCs. Hout et al. [112] synthesized CuS nanoparticles anchored on rGO nanosheets by using the hydrothermal method. The specific surface area of CuS/rGO was approximately 34.4 m^2^ g^−1^ and the volume of the swollen pores was 0.0595 cm^3^ g^−1^. Its specific capacitance reached 587.5 F g^−1^ at a current density of 1 A g^−1^, and its retention rate was 95% after 2000 cycles at a current density of 10 A g^−1^. Boopthiraja et al. [113] prepared hexagonal CuS/rGO nanocomposites by using the hydrothermal method in which hexagonal CuS grains were uniformly distributed on the rGO surface. The composite exhibited a large specific surface area of 122 m^2^ g^−1^ and pores of size 8–10 nm. The specific capacitance was 1604 F g^−1^ at a current density of 2 A g^−1^, and the capacitance was maintained at 97% of the initial level after 5000 cycles. In addition to the hydrothermal method, the composite of graphene and copper–sulfur compounds prepared using successive ionic layer adsorption (SILAR) has been reported. Bulakhe et al. [114] used the SILAR method to modify Cu_2_S nanosheets to prepare a nanocomposite Cu_2_S/rGO electrode. This nanohybrid exhibited the specific capacitance of 1293 F g^−1^ at a scan rate of 5 mV s^−1^, which is higher than those of Cu_2_S (761 F g^−1^) and rGO (205 F g^−1^), with the capacity retention of 94% after 10,000 cycles. Malavekar et al. [115] used the SILAR method to deposit rGO and CuS nanoparticles on a flexible stainless steel substrate in successive layers to obtain CuS/rGO composites with a layered porous structure. The composites have a specific surface area of 77 m^2^ g^−1^ and an average pore size of 22 nm. Their specific capacitance reached 1201.8 F g^−1^ at a scan rate of 5 mV s^−1^, and the capacity was maintained at 98% after 3000 cycles. In our group, composites of copper sulfide compounds and rGO were prepared using the continuous ionic layer adsorption method, which exhibited a specific capacitance of 355.40 F g^−1^ at a current density of 0.5 A g^−1^.

Graphene-based copper–sulfur compound composites show excellent electrochemical behavior in SC applications; however, they have some drawbacks such as low electrochemical stability. Moreover, the oxidation state of copper is prone to disproportionation under normal experimental conditions, causing complexity of the material composition. Additionally, the surface agglomeration of nanomaterials and the resistance at the electrode–electrolyte interface are high, and a weak bonding force between graphene and metal sulfide nanomaterials leads to electrode shedding and rapid degradation. Sc-related data of copper-sulfur composites and graphene composites are listed in Table 4.

#### 6.3.2. Copper–Sulfur Composite with Carbon Nanotubes for SC Applications

Carbon nanotubes (CNTs) have a one-dimensional nanostructure, that is, a hexagonal network of tubular structures bonded by carbon material SP^2^. Because of their high electrical conductivity, excellent thermal and mechanical properties, light weight, large surface area, and unique pore structure, they can effectively improve the energy storage capacity and are widely applied for SC-active electrodes. Zhao et al. [127] prepared carbon dot (CQD)-modified CuS/CNTs composites with a three-dimensional grapevine-string-like structure by using the hydrothermal method at 180 °C for 12 h. The diameters of the CuS spheres were in the range 1–2 μm, and the doping of CQDs led to the reduction in the diameter of the CuS spheres to 100–500 nm, which caused the CuS spheres to interact closely with CNTs and accelerated the diffusion of electrons and ions. Their capacitance was 736.1 F g^−1^ at a current density of 1 A g^−1^, with the capacitance retention of 92% after 5000 cycles. The unique three-dimensional (3D) grapevine structure and the synergistic effect of CuS, CNTs, and CQDs provided additional pseudocapacitance and shortened the diffusion pathway. Quan et al. [128] synthesized 3D porous hierarchical CuS/CNTs@NFs with a flower-like morphology on nickel-foam-based carbon nanotubes by using the solvent-thermal method. CuS/CNTs were uniformly dispersed over the nickel foam surface, and the specific capacitance of 467.02 F g^−1^ was achieved at 0.5 A g^−1^, which is higher than those of CuS/CNTs (173.84 F g^−1^) and CuS (163.51 F g^−1^). The crosslinked structure of the composites provided a fast and easy path for charge transfer while effectively suppressing the self-coiling and agglomeration of CuS nanosheets, which resulted in a higher stability of the composite during the charging and discharging processes.

Improvements in the properties of nanocomposites based on CNTs are attributed to their unique morphology and the synergistic effects of the components, which increase the surface area, thus providing more active sites for electrolyte ions. In addition, CNTs, as the skeleton of highly conductive nanometers, accelerate the charge transfer process and provide a buffer matrix to effectively regulate volume changes under several rapid charge and discharge cycles. However, the current performance of copper–sulfur compound nanocomposites based on CNTs is far lower than the theoretical value, and the agglomeration problem on the surface of nanomaterials adversely affects the electrochemical performance. SC-related data on copper–sulfur composites with carbon nanotubes are listed in Table 5.

#### 6.3.3. Copper Sulfide Composite with Activated Carbon in SC

Activated porous carbon (PPAC) has been widely used because of its excellent electrochemical properties, low cost, and large specific surface area [134]. Most of its micropores have diameters between 2 and 50 nm and surface areas up to 3000 m^2^ g^−1^. Li et al. [135] used a solvent-thermal method to homogeneously grow 3D CuS microflora consisting of stacked nanosheets on PPAC. The specific capacitance of the CuS/PPAC electrode was 954.0 F g^−1^ at a current density of 1.0 A g^−1^, higher than those of pure CuS (579.2 F g^−1^) and PPAC (329.6 F g^−1^). The energy density of CuS/PPAC was 47.70 Wh kg^−1^, which is nearly double that of CuS (29.08 Wh kg^−1^). The capacitance retention after 5000 charge/discharge cycles was 81.99%, which was higher than that of pure CuS-based electrodes (60.59%). Wang et al. [136] prepared porous CuS/AC with a three-dimensional hollow flower-like structure by using the solvent-thermal method. The specific surface area of the CuS/AC composite was as high as 539.34 m^2^ g^−1^ and the pore volume reached 0.22 cm^3^ g^−1^. The high specific surface area and mesoporous structure mitigated the capacity decay because of the volume change during charging and discharging and promoted the diffusion pathways for ionic conductivity and electrolyte penetration, as well as increased the active reaction sites between the electrolyte and electrodes. The introduction of the PPAC layer reduced the electrical resistance from 0.69 Ω for CuS to 0.31 Ω for CuS/AC, which improved the surface contact between CuS and the electrolyte and enhanced diffusion performance. The specific capacitance was 247 F g^−1^ at a current density of 0.5 A g^−1^, and the capacitance retention was 92% after 5000 cycles.

#### 6.3.4. Copper–Sulfur Compounds Compounded with CC in SC

CC is a carbon-based material woven from carbon fibers that exhibits characteristics such as a light weight, low cost, high strength, low density, low thickness, and excellent flexibility. Gong et al. [137] grew CuS on CC in situ through chemical plating, achieving a specific capacity of 1387.1 F g^−1^ at a current density of 2 A g^−1^, and a capacity retention of 82.9% after 10,000 charge/discharge cycles. Zhou et al. [36] used the solvothermal method to grow dense CuS nanosheets on CC. The staggered ortho-hexagonal CuS nanosheets with suitable channels between them provided abundant electrochemically active sites and facilitated carrier exchange between the electrolyte and electrode interfaces. CuS generated after 4 h of the solvothermal reaction achieved the lowest impedance value (0.84 Ω) and a specific capacity of 436.5 mF cm^−2^ at a current density of 1 mA cm^−2^, which is higher than those of CC (2.36 mF cm^−2^), CuS/CC-3 (407.7 mF cm^−2^), and CuS/CC-5 (430.5 mF cm^−2^). The capacitance retention of the electrolyte after 5000 charge/discharge cycles was 75.1%. Jin et al. [138] deposited CuS nanosheets on conductive mesoporous CC through electrodeposition. The conductive CC served as both the current carrier and the skeleton of the composite. The g-CuS/CC and p-CuS/CC electrodes were prepared through the constant current and constant potential deposition methods, respectively, with g-CuS/CC achieving a specific surface area of 450.76 m^2^ g^−1^, which was larger than those of p-CuS/CC (397.84 m^2^ g^−1^) and CC (380.46 m^2^ g^−1^). At a current density of 2 mA cm^−2^, g-CuS/CC exhibited the capacitance of up to 4676 mF cm^−2^, higher than that of p-CuS/CC (3527 mF cm^−2^) and 10 times that of CC (490 mF cm^−2^), with a capacity retention of 89.8% after 10,000 cycles.

The conductive CC acts as a skeleton framework for the electrodeposited composite as well as a collector for the electroactive material. This unique manufacturing process makes the interface extremely smooth while realizing electrochemical double-layer capacitor and pseudocapacitor energy storage, resulting in enhanced electrochemical performance.

#### 6.3.5. Copper–Sulfur Composite with Acetylene Black in SC

Acetylene black (AB) is a carbon material produced through the carbonization of acetylene by controlled combustion under pressure in air and has attracted much attention in the field of energy storage because of its light weight, low specific gravity, strong electrolyte absorption ability, chemical stability, low cost, and excellent electrical conductivity [139,140,141]. Huang et al. [142] used the solvothermal method to synthesize AB CuS nanosheet composite CuS/AB with a laminated structure. The intensity ratio of D-band to G-band ID/IG was 1.34, higher than that of pure AB (1.12), resulting in the generation of more defects and vacancies, an increase in the interfacial area between electrolyte/electrode, and enhanced electron transfer. The high conductance of AB and the short ion diffusion paths in the layered CuS nanosheets resulted in a CuS/AB specific capacitance of 2981 F g^−1^ at a current density of 1 A g^−1^, higher than those of pure CuS nanosheets (920 F g^−1^) and AB (658 F g^−1^), and the specific capacitance of the CuS/AB was considerably higher than that of pure AB (1.12). After 600 cycles, the capacity of CuS/AB was maintained at 92%, whereas the CuS and AB electrodes remained at 72.5% and 47.7%, respectively. The specific surface area of pure CuS nanosheets increased from 16.75 m^2^ g^−1^ to 62.37 m^2^ g^−1^, which increased the interface area between the electrolyte and electrode. Moreover, the high conductivity of AB and the CuS layer shortened the ion diffusion path and promoted electron transfer. AB anchored on CuS nanosheets to form a stable three-dimensional structure, reduces deformation, avoids the destruction of electrode materials, and maintains good stability during charge and discharge cycles.

#### 6.3.6. MOF-Derived Copper–Sulfur Compound/Carbon-Based Nanocomposites for SC Applications

Metal organic frameworks (MOFs) are three-dimensional inorganic/organic hybrid materials with a periodic network structure formed by the self-assembly of transition metal ions and organic ligands. Generally, with metal ions as the connecting point and organic ligands as a support, MOFs exhibit a large specific surface area, high porosity, low density, tailorability, tunable specific surface area, abundant pores, and structural diversity. MOFs are used as templates for special materials and precursors used to fabricate high porosity. The poor electrical conductivity and cyclic stability of MOF-derived porous materials make it difficult to obtain a high-performance SC when used as electrode materials. Therefore, combining the MOF-derived materials with other materials to form a composite electrode material for improving conductivity, specific capacity, and stability remains challenging. Cu-BTC[Cu_3_(C_9_H_3_O_6_)_2_(H_2_O)_3_]_n_, also known as HKUST-1, is a stable porous MOFs material. Wu et al. [143] used HKUST-1 as a template to prepare carbon-coated Cu_1.96_S in a single run by using the vulcanization method, which converted 10 nm ultrafine Cu_1.96_S nanoparticles uniformly embedded in octahedral porous carbon into porous Cu_1.96_S/C composites and maintained the octahedral morphology of MOFs during vulcanization and carbonization. Cu_1.96_S/C exhibits a large specific surface area (140.4 m^2^ g^−1^), which provides more active sites, and it has higher stability than Cu_1.96_S/C at a current density of 0.5 A g^−1^, a specific capacitance of 200 F g^−1^, and a capacity retention of 80% after 3000 constant current charge/discharge cycles. Niu et al. [144] prepared flexible composite electrodes, CuS/CNTs, by connecting HKUST-1-derived CuS polyhedra with CNTs. The uniformly distributed CuS polyhedra, consisting of a number of nanorods, had a large specific surface area of 126.4 m^2^ g^−1^ and excellent pore size distribution, resulting in a high specific capacity of 606.7 F g^−1^ at 1 A g^−1^, with the capacitance being 87.0% of the initial value after up to 6000 cycles at 5 A g^−1^. SC-related data on metal organic skeleton-derived carbon copper sulfide-based nanocomposites are listed in Table 6.

### 6.4. Copper–Sulfur Compounds Compounded with Conductive Polymers for SC Applications

Conductive polymers are those whose conductivity is adjusted in accordance with those of semiconductors and conductors through doping and other means. The conductivity of the doped conductive state polymers reached more than 103 S cm^−1^ and compounding them with copper–sulfur compounds could enhance the conductivity of the electrodes and improve the performance of supercapacitors. Conductive polymer composites with copper–sulfur compounds can increase the effective surface area of the electrode material, reduce the penetration of the electrolyte and the side reaction with the electrode, and improve the stability and specific capacitance of the electrode material. Conductive polymers exhibit excellent flexibility and plasticity and can make the electrode material more flexible and deformable to facilitate adaptation to changes in shape and size of the electrode requirements. A typical conjugated polymer is polypyrrole (PPy), which is a promising SC material because of its high electrical conductivity, thermal and environmental stability, ease of synthesis, and nontoxicity. Liu et al. [146] prepared flexible polyester/copper sulfide/polypyrrole composite electrodes (PET/CuS/PPy) by using insulating polyester fabrics as a substrate material. CuS in the form of microspheres was generated on the surface of the modified PET fabric through chemical deposition to provide more PPy electrodeposition loading sites. The impedance value of the PET/CuS/PPy electrode was 0.35 Ω, which is considerably smaller than those of pure PPy (1.21 Ω) and PET/CuS (0.98 Ω). Moreover, it exhibited a high specific capacitance of 5410 mF cm^−2^ at 2 mA cm^−2^. CuS microspheres provide a stable structure during the charge/discharge cycle, whereas the PPy coating inhibited the detachment of CuS microspheres and promoted electron transport. Their capacitance remained at 84.1% of the original value after 2000 constant current charge/discharge cycles, which was superior to the 64.1% of the PET/CuS electrode and the 57.8% of the PPy electrode. Peng H. et al. [147] reported on the CuS nanosphere composite electrode material coated with PPy. Because of the synergistic effect of PPy and CuS, the high conductivity of CuS, and the short ion diffusion paths in porous spherical CuS, a high specific capacitance of 427 F g^−1^ was achieved at a current density of 1 A g^−1^, and the capacity retention rate was 88% after 1000 cycles. Peng S. et al. [148] successfully deposited PPy and CuS on a bacterial cellulose membrane (BC) and prepared flexible PPy/CuS/BC nanocomposite electrodes. The specific capacitance was up to 580 F g^−1^ at a current density of 0.8 mA cm^−2^.

PANI is another promising conducting polymer for energy storage with high specific capacitance and high electronic conductivity (30–200 S cm^−1^). Liu et al. [149] prepared CuS/C electrode material with a dense cloud-like structure by using a two-step hydrothermal method at 120 °C. The electrode exhibited an excellent microstructure, that is, a regular flocculated network, no agglomeration, and a smooth surface, with gaps between uniform nanoparticles. The nanocomposite CuS/C@PANI was prepared by depositing PANI on the CuS/C electrode, which helped achieve a specific capacitance of 425.53 F g^−1^ (1 A g^−1^) in 3 M KCl and a capacitance retention of 89.86% after 3000 cycles. The synergistic effect of various components of the CuS/C@PANI electrode led to the generation of abundant active sites for electrochemical reactions, promoting the diffusion and transfer of electrolyte ions during the electrochemical reaction, and the incorporation of the conductive polymer PANI resulted in excellent cycling stability and multiplicity performance.

Although conductive polymers have been found to promote faster charge transport kinetics and pseudocapacitance storage in nanocomposites, these polymers have some shortcomings. For example, polyaniline and polypyrrole have a high specific capacity but a low voltage window, whereas monomers have certain toxicity. Polythiophene and its derivatives have lower relative biological toxicity but lower capacity. Processing of conductive polymers is also challenging, and their energy storage process is accompanied by the doping and dedoping of ions, with the repeated entry and exit of ions on the polymer chain leading to the fracture of the molecular chain as well as the generation of irreversible capacity, eventually resulting in its instability in the long-term cycle.

## 7. Summary and Expectation

The present review focused on the application and research progress of copper–sulfur compounds in SCs. It discusses the effects of different stoichiometric ratios and the morphology of copper–sulfur compounds on SC properties in detail. Among copper–sulfur compounds with different stoichiometric ratios, CuS has been reported as the most common SC electrode material, with the highest specific capacitance among copper–sulfur compounds. A porous structure with a large specific surface area in different morphologies of copper–sulfur compounds increases the chemical active site of redox reaction charge storage, thus increasing the redox efficiency. The three-dimensional multistage structure can shorten the diffusion distance of ions and electrons and enhance the charge transport efficiency. The inner cavity of the hollow nanostructure can buffer the volume expansion and adapt to the volume change of the copper–sulfur compound in the long-term charge and discharge process, resulting in improved service life. With optimization of the preparation method, the structure and morphology of the copper–sulfur compound were regulated to optimize its specific properties. The selection of a conductive substrate with high conductivity and no use of additional adhesive reduce contact resistance, enhance ion transport, and improve the electrochemical performance. To solve the problems of the agglomeration and poor stability of copper–sulfur compound electrodes, nanocomposite materials with a large specific surface area and uniform pore distribution were synthesized by combining copper–sulfur compounds with other materials, which could effectively improve the specific capacitance and stability of copper–sulfur compound SCs. This review also discusses the electrochemical properties of copper–sulfur compounds with various transition metals, carbon-based materials, and conductive polymer composites, and highlights the benefits and functional role of each component in improving the overall electrochemical response. For the development trend of copper–sulfur compound supercapacitors, we should pay attention to several aspects: (1) structure: the three-dimensional multistage nanostructure and three-dimensional hollow structure of copper–sulfur compound composite nanomaterials have better performance; (2) preparation method: at present, the hydrothermal method and deposition method are used more, but it is also necessary to determine the appropriate method according to the actual situation of the experiment; and (3) the composite material characteristics: with good stability, good electrical conductivity, large specific surface area, and other characteristics, such as graphene, etc. Recently, MXenes have received wide attention and popularity. MXenes, similar to graphene, have the characteristics of a high specific surface area and high conductivity, and also have the advantages of being flexible and adjustable components. They can be embedded by polar organic molecules and metal ions into various univalent/polyvalent cations, showing a higher volume capacity than commercial double electric layer capacitors. However, there is no relevant research on the application of copper–sulfur compounds and MXene composites in SC.

Continued research efforts have led to the development of an SC system with a copper–sulfur compound as the electrode; however, the performance required for commercial application has not been achieved yet. Copper–sulfur compound supercapacitors still face many challenges, which are as follows: copper–sulfur compounds have distinct chemical calculation ratios, and the specific capacitance of copper–sulfur compounds varies with their stoichiometric ratios. Among them, CuS has the highest ratio; however, most of the reported preparation methods cannot accurately control the stoichiometric ratio, often resulting in more than two copper–sulfur compound mixtures. Therefore, new preparation methods need to be optimized for accurately controlling the stoichiometric ratio. Moreover, the existing preparation methods need to be studied in detail, so as to better control the morphology, specific surface area, pore size, and porosity of copper–sulfur compounds. Some studies have begun using first principles calculations to predict the properties and synthesis paths of metal sulfides, but theoretical calculations and simulation techniques for copper–sulfur compounds remain understudied, and there is still a huge space for development. Some copper–sulfur compound composites with other nanomaterial composite electrodes have higher specific capacity; however, their cyclic stability is low. It is necessary to further explore the relationship between the electrochemical behavior of copper–sulfur compounds and their structural characteristics, especially surface characteristics, porosity, and crystal phase. Notably, copper–sulfur compounds used as electrode materials, due to volume expansion in the application process, result in poor cyclic stability of SCs, and the current research on electrode loss is in its nascent stage. Given the lack of in-depth characterization, it is necessary to study the causes, mechanisms, and solutions to overcome the problem of the poor stability of copper–sulfur compound SCs.

## Figures and Tables

**Table 1:** Characteristics of supercapacitors with CuS as the electrode.

NO.	Electrode Material	Measurement Type	Operating Window (V)	Electrolyte	Energy Storage Performance	Retention Rate	Refs
1	CuS nanoplatelets	Three-electrode	−0.40~0.30	1 M LiClO_4_	72.85 F g^−1^ (3 A g^−1^)	-	[35]
2	CuS nanoflakes	Three-electrode	−0.90~0.20	3 M KOH	436.5 mF cm^−2^ (10 mA cm^−2^)	75.1% after 5000 cycles	[36]
3	CuS nanowire array	Three-electrode	0.00~0.50	1 M NaOH	305 F g^−1^ (0.6 mA cm^−2^)	87% after 5000 cycles	[37]
4	Flower-like CuS	Three-electrode	−1.10~0.40	2 M KOH	597 F g^−1^ (1 A g^−1^)	80% after 1000 cycles	[38]
5	CuS nanoparticles	Three-electrode	−0.20~0.70	1 M NaOH	101.34 F g^−1^ (1.5 mA cm^−2^)	81% after 1000 cycles	[39]
6	CuS	Three-electrode	0.00~0.50	2 M KOH	237 F g^−1^ (0.5 A g^−1^)	88% after 4000 cycles	[40]
7	CuS nanosheets	Three-electrode	−0.40~0.60	6 M KOH	833.3 F g^−1^ (1 A g^−1^)	75.4% after 500 cycles	[29]
8	Three-dimensional CuS nanowalls	Three-electrode	−0.30~0.60	2 M KOH	1124 F g^−1^ (15 mA cm^−2^)	90.7% after 2000 cycles	[41]
9	CuS spherical clusters	Three-electrode	0.00~0.50	3 M KOH	713 F g^−1^ (1 A g^−1^)	73% after 1000 cycles	[42]
10	Nanostructured CuS networks	Three-electrode	0.00~0.50	2 M KOH	49.8 mAh g^−1^ (1 A g^−1^)	80.5% after 1500 cycles	[43]
11	Three-dimensional CuS microflower	Three-electrode	0.00~0.50	2 M NaOH	438.0 F g^−1^ (3 mA cm^−2^)	87% after 2000 cycles	[44]
12	core–shell CuS@CQDs	Three-electrode	−0.10~0.50	6 M KOH	618 F g^−1^ (1 A g^−1^)	95% after 4000 cycles	[45]
13	CuS	Three-electrode	0.00~0.45	2 M KOH	718.48 F g^−1^ (2 A g^−1^)	89.2% after 3000 cycles	[46]
14	CuS	Three-electrode	−0.20~0.50	2 M KOH	298 F g^−1^ (2 A g^−1^)	100% after 2000 cycles	[47]
15	CuS thin films	Three-electrode	−0.40~0.80	1 M NaOH	132 F g^−1^ (50 mA cm^−2^)	-	[48]
16	CuS thin films	Two-electrode	−1.50~1.00	1 M NaOH	102 F g^−1^ (10 mV s^−1^)	-	[49]
17	CuS nanoparticles	Three-electrode	−0.20~0.60	3 M KOH	164.053 mAh g^−1^ (1 A g^−1^)	97.12% after 4000 cycles	[50]
18	CuS@CQDs	Three-electrode	−0.10~0.50	6 M KOH	920.5 F g^−1^ (0.5 A g^−1^)	92.8% after 10,000 cycles	[51]
19	Hexagonal CuS	Three-electrode	−0.10~0.40	6 M KOH	1123 F g^−1^ (1 A g^−1^)	87% after 4000 cycles	[52]

Note: core–shell CuS@CQDs carbon quantum dots in the table represent hollow nanospheres of core–shell-coated CuS.

**Table 2:** SC-related data on the formation of polymetallic sulfide composites of copper–sulfur compounds with other metals.

NO.	Electrode Material	Measurement Type	Operating Window (V)	Electrolyte	Energy Storage Performance	Retention Rate	Refs.
1	CuCo_2_S_4_/ rGO	Three-electrode	0.01~0.60	3 M KOH	525 F g^−1^ (1 A g^−1^)	83% after 1000 cycles	[85]
2	CuCo_2_S_4_/ CNT/GO	Three-electrode	0.00~0.40	6 M KOH	504 F g^−1^ (10 A g^−1^)	92.3% after 2000 cycles	[86]
3	CuCo_2_S_4_ hollow nanoneedle arrays	Three-electrode	−0.10~0.50	3 M KOH	2163 F g^−1^ (6 mA cm^−2^)	98.7% after 6000 cycles	[87]
4	CuCo_2_S_4_	Three-electrode	0.00~0.45	4 M KOH	516 F g^−1^ (10 A g^−1^)	66% after 10,000 cycles	[88]
5	CuCo_2_S_4_	Three-electrode	0.00~0.50	3 M KOH	3321.6 F g^−1^ (5 A g^−1^)	87.1% after 3000 cycles	[89]
6	CuCo_2_S_4_	Three-electrode	0.00~0.45	6 M KOH	1839 F g^−1^ (5 A g^−1^)	85.3% after 2000 cycles	[90]
7	CuCo_2_S_4_/ CNT	Three-electrode	0.00~0.45	1 M KOH	1690.3 F g^−1^ (1 A g^−1^)	95.5% after 10,000 cycles	[91]
8	Cu_2_MoS_4_	Three-electrode	−0.80~0.20	1 M Na_2_SO_4_	127 F g^−1^ (1.5 mA cm^−2^)	91.78% after 3000 cycles	[92]
9	CuCo_2_S_4_	Three-electrode	0.00~0.45	6 M KOH	2446.6 F g^−1^ (1 A g^−1^)	82% after 10,000 cycles	[93]
10	CuCo_2_S_4_	Three-electrode	0.00~0.45	1 M KOH	592 F g^−1^ (1 A g^−1^)	90.4% after 3000 cycles	[94]
11	CuCo_2_S_4_	Three-electrode	−0.25~0.40	Polysulfide electrolyte	5030 F g^−1^ (20 A g^−1^)	79.5% after 2000 cycles	[95]

**Table 3:** SC-related data on copper–sulfur compounds complexed with other metal compounds.

NO.	Electrode Material	Measurement Type	Operating Window (V)	Electrolyte	Energy Storage Performance	Retention Rate	Refs
1	CuS/rGO/ Ni_3_S_2_	Three-electrode	−0.10~0.45	6 M KOH	1692.7 F g^−1^ (6.5 A g^−1^)	91.5% after 4000 cycles	[103]
2	Ni_3_S_4_/CuS	Three-electrode	0.00~0.45	6 M KOH	1917 F g^−1^ (1 A g^−1^)	91.2% after 3000 cycles	[104]
3	3D/2D Cu_2_Se /CuS	Three-electrode	0.00~0.40	6 M KOH	2727 F g^−1^ (2.5 mA cm^−2^)	70.2% after 8000 cycles	[105]
4	Cu_x_S_y_/CoS_z_/GO	Three-electrode	0.00~0.55	2 M KOH	324 F g^−1^ (1 A g^−1^)	-	[106]
5	Ni_3_S_4_/CuS	Three-electrode	0.00~0.60	2 M KOH	888 F g^−1^ (1 A g^−1^)	83.33% after 2000 cycles	[107]
6	CuS/ZrO_2-_	Three-electrode	0.00~0.50	2 M KOH	949.47 F g^−1^ (5 mV s^−1^)	-	[108]

**Table 4:** SC-related data for copper–sulfur composites with graphene composites.

NO.	Electrode Material	Measurement Type	Operating Window (V)	Electrolyte	Energy Storage Performance	Retention Rate	Refs
1	CuS/rGO	Three-electrode	−0.90~0.10	2 M KOH	368.3 F g^−1^ (1 A g^−1^)	88.4% after 1000 cycles	[110]
2	CuS/GO	Two-electrode	0.00~1.00	3 M KOH	197.45 F g^−1^ (5 mV s^−1^)	90.35% after 1000 cycles	[111]
3	CuS/rGO	Three-electrode	0.00~0.40	6 M KOH	587.5 F g^−1^ (1 A g^−1^)	95% after 2000 cycles	[112]
4	CuS/rGO	Three-electrode	0.00~0.50	3 M KOH	1604 F g^−1^ (2 A g^−1^)	97% after 5000 cycles	[113]
5	Cu_2_S/rGO	Three-electrode	−1.00~0.00	1 M KOH	1293 F g^−1^ (1 A g^−1^)	94% after 10,000 cycles	[114]
6	CuS/rGO	Three-electrode	−1.10~−0.20	1 M LiClO4	1201.8 F g^−1^ (5 mV s^−1^)	98% after 3000 cycles	[115]
7	CuS/rGO	Three-electrode	−0.20~0.40	6 M KOH	2317.8 F g^−1^ (1 A g^−1^)	96.2% after 1200 cycles	[116]
8	CuS@CQDs-GOH	Three-electrode	−0.10~0.50	6 M KOH	920 F g^−1^ (1 A g^−1^)	90% after 5000 cycles	[117]
9	CuS/GO	Three-electrode	0.00~0.58	3 M KOH	249 F g^−1^ (4 A g^−1^)	95% after 5000 cycles	[118]
10	CuS/rGO	Three-electrode	0.00~0.55	3 M KOH	203 F g^−1^ (0.5 A g^−1^)	90.8% after 10,000 cycles	[119]
11	CuS/CN	Three-electrode	−0.80~1.00	0.1 M Li_2_SO_4_	379 F g^−1^ (1 A g^−1^)	72.46% after 500 cycles	[120]
12	CuS/GO	Three-electrode	−0.80~−0.15	6 M KOH	497.8 F g^−1^ (0.2 A g^−1^)	91.2% after 2000 cycles	[121]
13	CuS/rGO	Two-electrode	0.00~1.00	6 M KOH	906 F g^−1^ (1 A g^−1^)	89% after 5000 cycles	[122]
14	CuS/rGO	Three-electrode	−0.20~0.60	2 M KOH	1222.5 F g^−1^ (1 A g^−1^)	91.2% after 2000 cycles	[123]
15	CuS/GO	Three-electrode	0.00~0.60	3 M KOH	250 F g^−1^ (0.5 A g^−1^)	70% after 5000 cycles	[124]
16	CuS/rGO	Three-electrode	−1.00~0.00	2 M KOH	3058 F g^−1^ (1 A g^−1^)	60.3% after 1000 cycles	[125]
17	Cu_2_S/rGO	Three-electrode	−0.20~−0.45	3 M KOH	1918.6 F g^−1^ (1 A g^−1^)	95.4% after 5000 cycles	[126]

Note: CuS@CQDs-GOH denotes the complex of carbon-dot-modified CuS with graphene oxide hydrogel; CuS/CN denotes the complex of nitrogen-doped graphene with CuS.

**Table 5:** SC-related data on copper–sulfur composites with carbon nanotubes.

NO.	Electrode Material	Measurement Type	Operating Window (V)	Electrolyte	Energy Storage Performance	Retention Rate	Refs
1	CuS/CNTs	Three-electrode	0.00~0.50	3 M KOH	736.1 F g^−1^ (1 A g^−l^)	92% after 5000 cycles	[127]
2	CuS/CNTs	Three-electrode	0.00~0.60	6 M KOH	467.02 F g^−1^ (0.5 A g^−1^)	86% after 5000 cycles	[128]
3	CuS/CNT	Three-electrode	0.00~0.50	2 M KOH	122 F g^−1^ (1.2 A g^−1^)	100% after 1000 cycles	[129]
4	CuS/CNTs	Three-electrode	−0.40~0.60	6 M KOH	2831 F g^−1^ (1 A g^−1^)	90% after 600 cycles	[130]
5	3D-CuS/CNTs	Two-electrode	0.00~0.60	2 M KOH	2204 F g^−1^ (10 mA cm^−2^)	89% after 10,000 cycles	[131]
6	CuS@CNT	Three-electrode	0.00~1.00	2 M KOH	1.51 F cm^−2^ (1.2 A g^−1^)	92% after 1000 cycles	[132]
7	CuS/CNTs	Three-electrode	−0.20~0.60	2 M KOH	566.4 F g^−1^ (1 A g^−l^)	94.5% after 5000 cycles	[133]

**Table 6:** SC-related data on metal organic skeleton-derived carbon copper sulfide-based nanocomposites.

NO.	Electrode Material	Measurement Type	Operating Window (V)	Electrolyte	Energy Storage Performance	Retention Rate	Refs
1	Cu_1.96_S/C	Two-electrode	0.00~0.90	1 M KOH	200 F g^−1^ (0.5 A g^−1^)	80% after 3000 cycles	[143]
2	CuS/CNTs	Three-electrode	0.00~0.50	6 M KOH	606.7 F g^−1^ (1 A g^−1^)	87.0% after 6000 cycles	[144]
3	Cu_1.8_S/C	Two-electrode	1.00~3.00	1 M LiPF_6_	740 mAh g^−1^ (50 mA g^−1^)	78% after 200 cycles	[23]
4	Carbon-coated Cu_7_S_4_	Three-electrode	−0.20~0.70	1 M H_2_SO_4_	321.9 F g^−1^ (0.5 A g^−1^)	78.1% after 3000 cycles	[22]
5	Cu_9_S_8_@C-CC@PPy	Three-electrode	−0.40~0.50	1 M KCl	270.72 F g^−1^ (10 mV s^−1^)	83.36% after 3000 cycles	[145]

Note: Cu_9_S_8_@C-CC@PPy denotes: MOF-derived carbon coated with CuS, followed by deposition of polypyrrole PPy on a carbon cloth substrate using electrochemical deposition.

## Data Availability

No new data is created.
